# Examination of the Compatibility of the Photogrammetric Method with the Phenomenon of Mora Projection in the Evaluation of Scoliosis 

**DOI:** 10.1155/2014/162108

**Published:** 2014-05-19

**Authors:** Justyna Drzał-Grabiec, Sławomir Snela, Justyna Podgórska-Bednarz, Justyna Rykała, Agnieszka Banaś

**Affiliations:** ^1^Institute of Physiotherapy, University of Rzeszów, 26 Warszawska Street, 35-205 Rzeszów, Poland; ^2^Department of Paediatric Orthopaedics and Traumatology, Regional Hospital, No. 2, 35-301 Rzeszów, Poland; ^3^Laboratory of Molecular Biology, Institute of Obstetrics and Medical Rescue, University of Rzeszów, 35-205 Rzeszów, Poland

## Abstract

*Introduction*. The aim of this study was to evaluate the compatibility of external measurements of parameters characterizing scoliosis using the photogrammetric method. *Material.* The study involved 120 children between the ages of 7 and 11 years in Podkarpackie (Poland). *Method*. Measurements of body posture characteristics were performed using the photogrammetric method with mora projection. Each person was examined twice, once by two different therapists, with a time lapse of 20 minutes in between examinations. *Results*. High accuracy and no statistical significance were found among different measurements of asymmetry parameters characterizing the shoulder blades and hips. Regularities were also found in the characteristic measurements of curves of scoliosis. The POSTI parameter showed a significant variation and lack of compatibility of results. *Conclusions*. (1) The photogrammetric method used to assess the pathological changes caused by scoliosis gives significant results in terms of parameters characterizing the position of the shoulder blades and shoulders, as well as pelvis rotation. (2) High compliance measurements are also characterized by the length of the right and left arcs of scoliosis.

## 1. Introduction

Scoliosis is a three-dimensional deformity of the spine affecting the formation and functioning of the musculoskeletal system [1–3]. Notably, the torsion of vertebrae is practically irreversible; therefore, early detection and treatment of scoliosis are extremely important. This prevents fixed structural changes, which may have consequences in adulthood in the form of back pain, curvature progression, psychosocial implications, and even disorders of the respiratory system [4–7]. Characteristics of scoliosis include abnormalities such as rib gibbosity, asymmetry of the costal arch, asymmetry of the waist, lumbar shift, distorted setting of the shoulder blades, and asymmetry of shoulders. These abnormalities lead to problems of body biomechanics, which could also impair the function of certain organs, as well as entire systems [7–9]. In clinical practice, diagnosis of scoliosis is based on X-ray examination where the Cobb angle value is calculated [10]. This result, however, does not provide a complete picture of the irregularities taking place in the body. In addition, radiographic exams are not innocuous to the human body; researchers are troubled with the repeated exposure carried out during X-ray examinations, especially in follow-ups [11, 12]. Radiographic examination enables imaging in two-dimensional space; therefore, there is no information on the consequences of three-dimensional spinal abnormalities [13, 14]. This is why numerous scientific reports are based on alternative methods in the assessment of scoliosis and its associated deformities. The obvious requirements of the assessment methods, in accordance with the requirements of the measurement tools, are that the method be fast, and that it demonstrates high reproducibility, compatibility, and reliability ratings. However, insofar, the information available on the use of assessment tools comes from both subjective and objective groups. It is known that the assessments of methods from subjective groups carry a wide margin of error, so, in many cases, they do not meet the needs of clinicians. To evaluate the parameters characterizing scoliosis, tools used included scoliometers, two- or three-dimensional radiography, and stable-metric platform end optoelectronic systems [15–18]. The most significant technique used was the photogrammetric method using the phenomenon of mora projection. The application of the Mora 4G system appears promising due to technical properties such as a very short response time, ease in preparation of the patient, and the possibility of recording an entire sequence of patient motion. This method provides many opportunities for data measurement, which may significantly facilitate the work of physiotherapists and physical rehabilitation professionals.

The aim of this study was to evaluate the compatibility of the external measurements of parameters characterizing scoliosis using the photogrammetric method with the phenomenon of mora projection.

## 2. Materials and Methods

The study involved 120 randomly selected children studying in primary schools in Podkarpackie (Poland). The age range of the study group was 7–11 years. Girls accounted for 58% of the study group and boys composed 42%. Prequalification of participants was to obtain guardian consent in writing. Both parents/guardians and children were informed of the possibility to withdraw at any time during the study. Exclusion criteria applied to the group included neurological deficits, mental disability impairing consistent posture, orthopedic disease, genetic musculoskeletal defects, congenital defects of the upper and lower limbs, and significant vision or hearing impairment.

**Figure 1:**
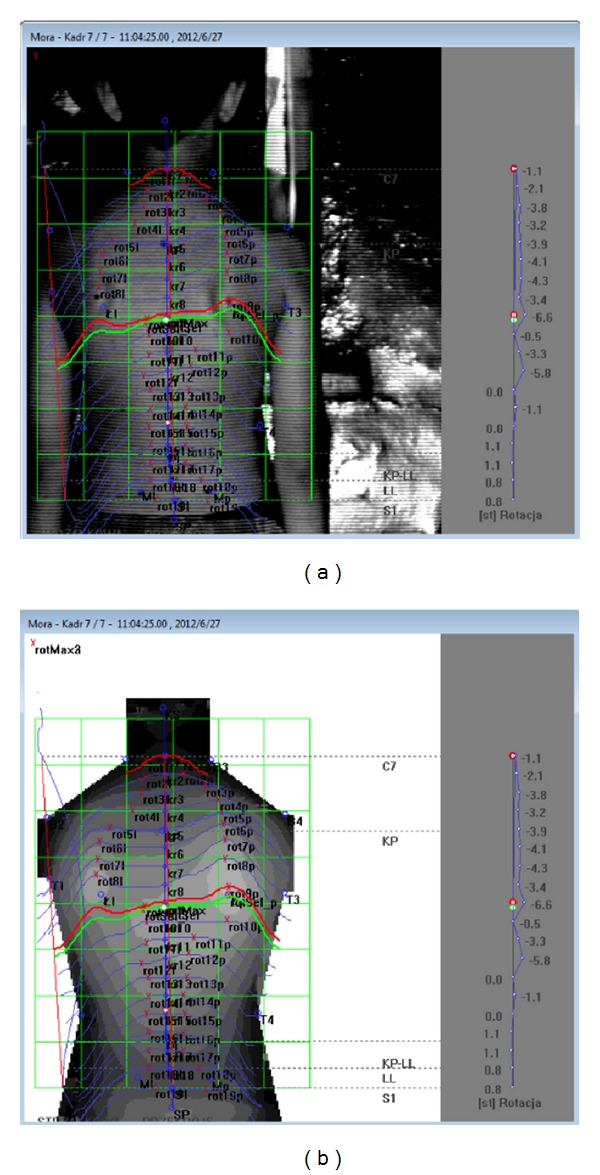
Imaging capabilities with the MORA 4th Generation system, by CQ Elektronik System company. Source: our images, obtained in our study.

The study was approved by the Bioethical Commission of the University of Rzeszow (number: 7/05/2012). The survey was conducted in May and June of 2012. The study took place at nursing clinics in selected educational institutions. For measurements of selected parameters characterizing posture, the photogrammetric method was applied using the phenomenon of mora projection (MORA System 4th Generation) (Figure 1). Each person was tested twice. The first measurement was performed by Evaluative I (individual therapist I), and the second examination was carried out 20 minutes later, performed by Evaluative II (individual therapist II). Each of individual therapists independently prepared the patient for the examination and performed the study and its analysis and interpretation. The study was performed in a relaxed standing position.

The parameters measured in the study were

 KNT: the angle of trunk declination, which determines the declination of the line C7-S1 from the vertical in the frontal plane KLB (mm),
 UL (mm): the difference in height of the lower blade angles (slope/inclination),
 UB (mm): the difference in the depth of the lower blade angles (torsion),
 OL: the difference in distance of the lower blade angles from the spine,
 KSM (mm): the angle of rotation of the pelvis,
 KLB (mm): the angle of the shoulder line,
 LpD: the length of right arc of scoliosis,
 LpK: the size of the angle of the right arc of scoliosis,
 LID: the length of left arc of scoliosis,
 LIK: the size of the angle of the left arc of scoliosis,
 POTSI: rear trunk asymmetry factor.

## 3. Statistics

The results were statistically analyzed using Statistica 7.1 (StatSoft Poland). In order to compare the results obtained with the evaluation of the two independent therapists, Spearman's rank correlation test and the Wilcoxon-signed rank test for sequences were used. Statistically significant results are highlighted in bold and marked with an asterisk (∗). Nonparametric tests were used due to noncompliance with the timetable of normal distribution. Normality of distribution was verified using the Shapiro-Wilk test. The accepted level of statistical significance was *P* < 0.05. Statistically significant results are highlighted in bold.

## 4. Results

Included in the evaluated parameters are parameters determining the correct position of the shoulder blades, shoulder line, pelvic rotation, and extent of scoliosis, such as the length of each arc of scoliosis and the value of an angle complementary to Cobb's angle; the smaller the angle, the more advanced the scoliosis (Table 1).

The first group of results are the values describing the position of the shoulder blades, that is, the difference in height of the lower blade angles (slope), the difference in the depth of the lower blade angles (twist), and the difference in distance of the lower blade angles from the spine. In all three cases, the high compliance measurements were obtained at *P* = 0.0000, and the differences between the two tests were not statistically significant. The smallest differences were for the UB parameter for which the level of *P* = 0.8667; the other two exceeded the value of *P* > 0.1.

The second value was the angle of the shoulder line, in which the compliance was also highly and statistically significant at *P* = 0.0000, while the differences exceeded the level of significance (*P* > 0.15). The difference between the averages obtained from all the measurements made by the two individual therapists was 0.83 mm.

The same compliance measurements and the difference were characterized by a parameter indicating the size of KSM (pelvic rotation in mm). This compatibility was *P* = 0.0000, size differences in the Wilcoxon test at *P* = 0.2199, while the size of the arithmetic difference was 0.31 mm.

In the group of parameters describing the size of the individual curves of scoliosis are the length of right arc of scoliosis (LpD), the size of the angle of the right arc of scoliosis (LpK) (the same parameters for the left arc), and rear trunk asymmetry factor (POTSI). For LpD and LID parameters, significant correlation was confirmed between the results obtained by two individual therapists. The correlation values for LpD and LID were *R* = 0.2 and *R* = 0.3, respectively. The difference between the averages for the measurements of the length of the right arc of scoliosis was 4.12 mm and, for the left arc, 18.57 mm, and they were irrelevant with respect to the Wilcoxon test. In the case of angular values for different curves, there was no correlation between the results obtained by individual therapists, and the value of the angle of the right arc was only slightly above the level of statistical significance (*P* = 0.547). The difference between values of averages was 0.2 mm for the angle of the right arc and 0.1 mm for the left arc. These values were, however, not statistically significant.

In the parameter of POTSI (rear trunk asymmetry factor), the correlation value of the results obtained in the evaluation of the two individual therapists was *R* = 0.2 at *P* = 0.006. The differences in the results obtained $\left(\left|{\overset{-}{x}}_{1}-{\overset{-}{x}}_{2}\right|=2.78\right)$ were statistically significant at *P* = 0.0025.

## 5. Discussion

Our findings show a pattern characterizing various subgroups of data. In the case of asymmetry parameters characterizing the shoulder blades and hips, high accuracy of the results was obtained with a lack of statistical significance with respect to differences between the assessments made by the two independent investigators. Another regularity was found in the characteristic quantities of the various curves of scoliosis and a global parameter for the body POSTI. The length of the individual arcs of scoliosis of both the right and the left was characterized by compatibility and lack of significant statistical differences in the tests obtained by the two therapists. The correlation of results for angular values, however, does not show compatibility, but there were no significant differences in tests. The POTSI parameter showed a significant variation and lack of compatibility of the results.

Our findings indicate the usefulness of the method used for the assessment of abnormalities caused by scoliosis and in the case of measurement, the length of each arc of scoliosis. The results are repeatable due to the fact that the photographic assessment is performed under specific conditions (i.e., constant distance, constant parameters of the optical system, carefully leveled camera position, body position set to “0” twist of the pelvis, and specific lighting conditions). The lack of conformation of the angle measurement of scoliosis is caused by the fact that photogrammetry is used for external measurements, so it is difficult to determine the precise location of the limit vertebrae used to determine the Cobb angle. However, measurements of angular curvature present no significant differences in our study, which shows that the method can be used in screening tests for the detection of scoliosis. Small discrepancies may be due to the fact that, in this study, it is necessary to determine photogrammetric anthropometric points where the measurement error is estimated to be ±5 mm. Another important factor is the accuracy of the examiner. In the photogrammetric survey, it is important to level the machine using the camera located on the level indicators, which, unfortunately, is often forgotten by researchers. Another possible error is the positioning of the characteristic points on the screen, which depends on the experience of the operator, the screen resolution in different planes, the size of the light spot, and the contrast of the selected points.

Saad Ruggeri et al. compared the results of the Cobb angle measurements obtained through the photogrammetric method and traditional radiographs. In examining the reliability and accuracy of the results, they concluded that the method, despite the high repeatability of results, cannot replace conventional radiography, which is currently the gold standard for the assessment of scoliosis. It is valuable, however, to use for confirmation of the validity of undertaken therapeutic actions, which certainly can reduce the number of radiographs performed within the entire period of treatment [19]. When used in our study, it was shown to give more accurate measurement results, partly due to automatic calculation of curvatures, which greatly reduces the size of error. In the Ruggeri et al. study on the reliability of photogrammetric methods on structural scoliosis, the authors showed good compatibility between the evaluators and the test-retest analysis. The Cobb angle values greatly affected the results achieved by Ruggeri et al. For higher values, higher external compatibility ratings were observed, especially in the case of trunk rotation evaluated from left side view [20]. In the studied group, no scoliosis present or only low-grade scoliosis was found. This may suggest that in the case of a group with more advanced scoliotic abnormalities, especially for parameters that are on the borderline of statistical significance, Cobb angle values may increase, favorably impacting the overall evaluation of the method used in this study. In the photogrammetric method, before measurement takes place, each investigator marks anthropometric points for later analysis and determination of specific parameters. It is obvious that in the case of more advanced scoliosis, it will be easier to determine the precise points and therefore to expect a greater compatibility result. A completely different relationship was obtained by Thometz, who compared the measurement made by the Quantec method with Cobb angle values. In this case, he achieved a greater correlation between the measurements for low-grade scoliosis [21]. The possible measurement error could also be due to the fact that the study using the photogrammetric method is unique, due to the lack of standardization for the position in which measurements are performed (i.e., the body position of a child, which deviates from its correct habitual position) [22]. What may be the reason for the lack of fully conclusive results of POSTI parameters, which is in the assessment made by Kotwicki et al., is characterized by high utility and its ability to replace the measurement of 11 other parameters. It should be noted that Kotwicki et al. conducted their study on a group of people with severe progression of scoliosis [23].

In a study of Saad et al., by comparing the Cobb angle in photogrammetric and radiographic examinations, convergence of the results was demonstrated [24]. Another confirmation of the compatibility of the photogrammetric method with the radiographic method was the work of the same team of authors, who compared the angle of thoracic kyphosis and the angle of lumbar lordosis using both methods [25]. Similar studies were conducted by Leroux and Zabijek, where in 124 patients, they compared measurement results of thoracic kyphosis and lumbar lordosis using radiographic and photogrammetric methods. The results indicated a high correlation; the correlation for thoracic kyphosis was 0.89, while for the lumbar lordosis it was slightly lower at 0.84 [26]. Iunes [27] and Van Maanen et al. [28] confirmed the accuracy of photogrammetry as a method of assessing posture. Similarly, Braun and Amundson [29] evaluated photogrammetry as a good method for assessing posture.

According to the American Society for Photogrammetry and Remote Sensing [30], photogrammetry is the science of obtaining reliable information about the shape of objects, which can be measured and interpreted. One of the advantages of photogrammetry is its ability to record minor changes and deviations [31]. The photogrammetric method is used to assess body posture and provides more reliable information than visual evaluation [32–34]. Another advantage is its ability to store results in form of digital files. Through analysis of the results of our study and those of other researchers, there is a clear conclusion about the accuracy of photogrammetry as a method of assessing body posture. An important argument is the compatibility of photogrammetry with the radiographic method, which is currently considered the gold standard in the diagnosis of scoliosis. The photogrammetric method cannot replace radiography, but because of its repeatability, high compatibility with the radiographic method, and its lack of invasiveness, it is a useful method for screening. The screening study can detect low-grade scoliosis, even in children in whom previous deformities had gone undetected. Such early detection of scoliosis has important prognostic significance. Using the photogrammetric method, the therapist can also, without any risk to the patient, monitor the effects of therapy and repeat the test as often as necessary. The emergence of new methods, such as photogrammetry with mora projection phenomenon, makes it possible to minimize the performance of X-ray studies. If therapists would perform the photogrammetric test as a primary test, they could direct a smaller proportion of patients to radiographic examination.

## 6. Conclusion

1. The photogrammetric method using mora phenomenon imaging used to assess pathological changes caused by scoliosis gives significant results in terms of parameters characterizing the position of the shoulder blades and shoulder and pelvis rotation.
2. High compatibility of tests is also characterized by the length of the right and left arc of scoliosis.
3. In further consideration of the possible wider use of the photogrammetric method in clinics, it would be necessary to conduct a detailed comparative analysis of Cobb angle measurements.
4. Application of this method is appropriate for the screening and evaluation of multiple scoliotic changes.
5. In clinical practice, the photogrammetric method with mora phenomenon imaging allows fast measurement and reduction of patients' exposure to X-rays.

## Figures and Tables

**Table 1:** Summary of selected parameters characterizing body posture with results of statistical analysis.

Variables	Test I			Test II			Difference	Test spearmana		Test wilcoxona	
	${\bar{x}}_{1}$	*S*	Me	${\bar{x}}_{2}$	*S*	Me	$\left|{\bar{x}}_{1}\;-\;{\bar{x}}_{2}\right|$	*R*	*P*	*Z*	*P*
KNT (mm)	−0.89	1.37	−1.00	−0.77	1.32	−0.80	0.12	**0.4**	0.0000*	0.56	0.5752
KLB (mm)	−2.53	7.38	0.00	−3.36	8.33	−0.45	0.83	**0.4**	0.0000*	1.4	0.1579
UL (mm)	−1.70	6.21	0.00	−0.70	5.73	0.00	1	**0.5**	0.0000*	1.6	0.1042
UB (mm)	−3.64	7.86	−3.00	−3.45	7.75	−4.15	0.19	**0.5**	0.0000*	0.16	0.8667
OL (mm)	2.35	11.85	1.00	2.85	8.93	2.65	0.5	**0.6**	0.0000*	1.3	0.1925
KSM (mm)	−2.50	5.05	−2.30	−2.81	4.82	−3.00	0.31	**0.5**	0.0000*	1.2	0.2199
LpD (mm)	79.94	135.34	0.00	75.82	133.18	0.00	4.12	**0.2**	0.0351*	0.32	0.7421
LpK (degree)	53.75	77.91	0.00	47.56	70.74	0.00	6.19	0.2	0.0547	0.54	0.5875
LID (mm)	238.81	156.21	326.20	220.24	160.50	316.15	18.57	**0.3**	0.0017*	1.58	0.1139
LIK (degree)	**123.83**	78.29	173.90	118.31	82.09	174.85	5.52	0.1	0.2449	0.43	0.6669
POTSI	18.21	9.01	16.80	15.43	7.17	14.60	2.78	**0.2**	0.0060*	**3.03**	0.0025*

Source: our study.

*Statistically significant results.
